# Navigating the Complexities of Disseminated Histoplasmosis Diagnosis and Management in the Migrant Population: A Case Report

**DOI:** 10.7759/cureus.61434

**Published:** 2024-05-31

**Authors:** Abiodun M Akanmode, Fatima Anwer, Matthew Kalloo, Eldon Redwood

**Affiliations:** 1 Department of Medicine, Harlem Hospital Center, New York City, USA

**Keywords:** endemic mycosis, migrant health, antiretroviral, disseminated histoplamosis, hiv aids

## Abstract

Histoplasma capsulatum is a dimorphic fungus that grows in nature as a mold or in culture but converts to a small yeast during cellular invasion. While most histoplasmosis infections are primarily asymptomatic or mildly symptomatic, disseminated histoplasmosis is a relentlessly progressive granulomatous disease that can mimic other granulomatous diseases, such as tuberculosis, sarcoidosis or coccidioidomycosis, more so in the proper context of immunosuppression.

The current global migrant crisis, particularly the United States migrant crisis conversation is mostly socio-political; however, it also has a public health implication as exemplified by the case of a 35-year-old male who migrated from Haiti via Chile and Mexico to the United States. He presented with a four-day history of fever, generalized body aches, and cough. This case underscores the importance of entertaining a myriad of differentials and avoiding the tendency for anchoring, especially when initial therapy yields little clinical response.

## Introduction

Histoplasmosis capsulatum is a common mycosis endemic to the Americas with an estimated 32% positivity in skin testing and it is influenced by climate, soil geology, and animal habitation [1,2]. It also occurs worldwide in Africa, Asia, and Australia. The endemic areas for histoplasmosis in the United States include the Ohio-Mississippi River valleys extending into parts of northern Maryland, southern Pennsylvania, central New York, and Texas [3].

As a dimorphic fungus, histoplasmosis exists naturally as mold and is transmitted via inhalation of tiny spores (conidia). After inoculating the host, its composition changes to yeast cells, given the favorable host body temperature. After the initial pulmonary involvement, systemic involvement is postulated via hematogenous spread [4].

The clinical manifestation of histoplasmosis varies and depends on various factors such as age, degree of immunosuppression, and the size of the inoculum. Similarly, travel history and exposure from an endemic area are key to diagnosis. Despite the endemic nature of histoplasmosis, it is still underdiagnosed as many other diseases mimic it in presentation. Similarly, limitations encountered during testing with the various testing modalities also impact diagnosis and subsequent management. The acute infection is characterized by a myriad of symptoms (fever, cough, myalgias, chest pain, and malaise) compounded with relatable physical and radiological findings. Chronic cavitary histoplasmosis is associated with apical pulmonary cavities and progressive respiratory symptoms that are much more insidious in onset and very similar to those of tuberculosis (TB) and the progressive disseminated disease, though rare, has been associated with extremes of age, crude prolonged exposure, and immunocompromised status of hosts, particularly those with HIV/AIDS [4,5].

## Case presentation

A 35-year-old Haitian male who recently migrated to the United States presented to the Emergency Room (ER) with a two-day history of intermittent high-grade fever, generalized body aches, nonproductive cough, night sweats, and bilateral leg burning sensation. He had no significant medical history and denied any substance use, sick contact exposure, or any recent medication intake. He has two female sexual partners, lives in a migrant shelter, is currently unemployed but previously worked in a construction company in Haiti, and denies any occupational work exposure. He traveled to the United States via Chile and Mexico a week ago and he denied chest pain, weight loss, hemoptysis, neck stiffness, nausea, vomiting, skin rash, lower urinary tract symptoms, or penile discharge. 

He was tachycardiac at 130 and had a fever of 102.6 Fahrenheit in the emergency department. Physical examination showed pallor with bilateral non-tender cervical, axillary, and inguinal lymphadenopathy. Initial laboratory tests showed bi-cytopenia with atypical lymphocytosis and mild transaminitis (Table 1).

**Table 1:** Initial lab values WBC - white blood cells, ALK - alkaline phosphatase, AST - aspartate aminotransferase, ALT - alanine aminotransferase

Component	Patient value	Reference
Hemoglobin	7.8	14-18 g/dL
WBC	3.84	4.80-10.80
Atypical lymphocyte	9.0 %	<0 %
Platelets	355	150-450
ALK	77	40-129 µ/L
AST	52	<40 µ/L
ALT	25	<40 µ/L
Total protein	9.2	6.4-8.3 g/dL
Albumin	3.00	3.97-4.94 g/dL
HIV	Positive	Negative

Considering intermittent high-grade fever with chills and travel, further blood workups, including TB QuantiFERON, Legionella antigen, hepatitis B and C serology, cryptococcal antigen, dengue, malaria, and Zika PCR, were done, which were all negative. Atypical lymphocytosis and the high protein gap were further evaluated with HIV testing, which came out positive. The confirmatory HIV test was also positive with CD4 of 135 and viral load at 3.4 million.

**Figure 1:**
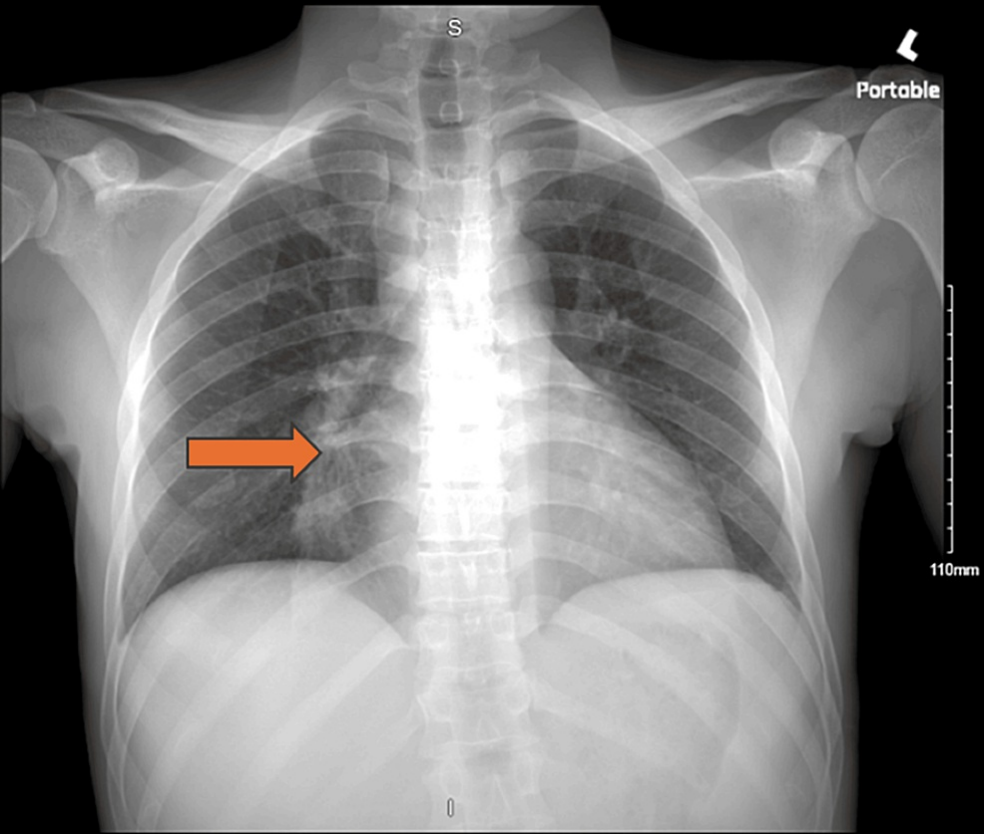
CXR showing right middle lobe infiltrate

**Figure 2:**
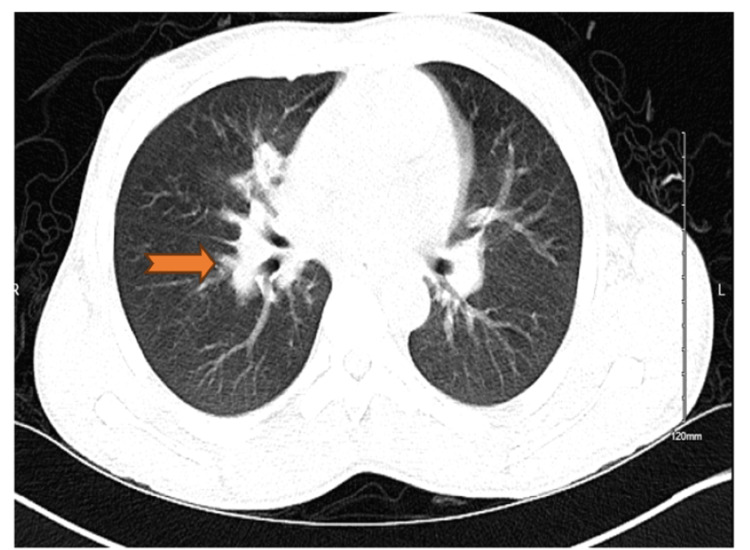
CT scan of the lungs showing right mid-lobe infiltrate

The initial chest x-ray showed possible consolidation of the right middle lobe (Figure 1). A follow-up non-contrast CT of the lungs revealed a small consolidation of the right middle lobe with an air bronchogram and bilateral axillary lymphadenopathy (Figure 2). CT of the abdomen showed hepatomegaly, splenomegaly, bilateral inguinal lymphadenopathy, and small free fluid in the pelvis.

Despite initiating treatment with Biktarvy (bictegravir-emtricitabine-tenofovir alafenamide) for HIV, Bactrim for Pneumocystis jirovecii pneumonia (PJP) prophylaxis, alongside empiric antibiotics (vancomycin, piperacillin-tazobactam, azithromycin) for suspected pneumonia sepsis, the patient continued to spike fever and showed no response. Fungitell beta-D-glycan assay utilized for suspected cases of invasive fungal disease was positive at 153 pg/mL. Considering his extensive travel history, a histoplasma urine antigen was conducted, which tested positive. We started empiric treatment for disseminated histoplasmosis. Subsequent histoplasmosis serology was negative, likely due to the already-initiated treatment. Cytomegalovirus (CMV) PCR was positive at 1,140; however, CMV pneumonitis was of low suspicion considering asymptomatic invasive tissue disease and CMV viremia being very common in HIV.

**Figure 3:**
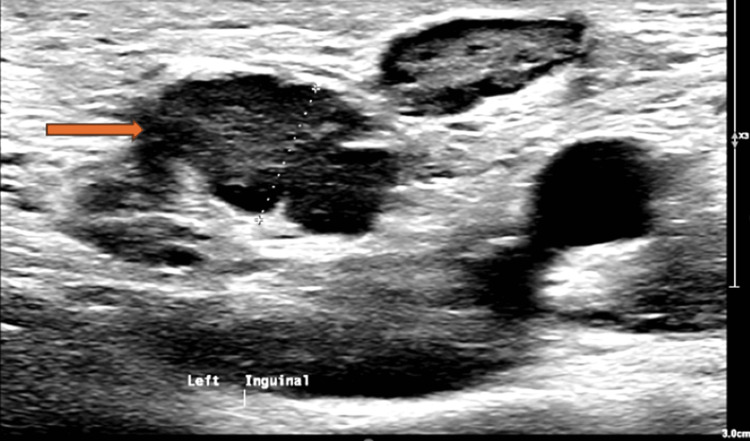
Left inguinal lymphadenopathy on ultrasound

The ultrasound-guided biopsy of the left inguinal lymph node (Figure 3) showed reactive lymphoid hyperplasia, with no lymphoma or carcinoma cells detected. Staining for microorganisms showed no visible organisms and no granulomas. Unfortunately, the fungal tissue culture could not be done as the sample was not collected.

The patient was initially started on IV amphotericin B, to which he responded, and broad-spectrum antibiotics were discontinued. After the complete resolution of the fever, Biktarvy was resumed, which was initially held temporarily due to concerns about immune reconstitution syndrome. He completed 10 days of Amphotericin B and was discharged home on itraconazole, Biktarvy, and Bactrim with a follow-up appointment in the ID virology clinic in one week; however, the patient failed to follow up.

## Discussion

The HIV pandemic has led to a surge of histoplasmosis cases in areas not known to be endemic [6]. Available data suggests that histoplasmosis is the most frequently imported mycosis, with cases ascribed to immigrants, expatriates, transient workers, or tourists who traveled to or from endemic countries and locations. Histoplasmosis is not a notifiable disease and is also not included in most public health surveillance systems; hence, it becomes a burden when disease quantification is attempted [7,8].

Our patient, like most migrants, was initially guarded about his migration history. However, after reassurance, we identified that he migrated through endemic areas such as Haiti, Chile, and Mexico to enter the United States. Further attempts to get collateral history about his travel details, such as specific migration routes, sick contacts exposure or exposure to bird droppings, were futile. While it is difficult to figure out exactly where and when he got infected, his concurrent diagnosis with HIV/AIDS makes him highly susceptible to disseminated histoplasmosis, and this is supported by studies from the 1950s that showed an intact immune system is essential in the prevention of disseminated histoplasmosis. However, immunocompromised patients are more prone, as evidenced by a higher number of cases of disseminated histoplasmosis among patients with Hodgkin's lymphoma [9].

The diagnosis of histoplasmosis can be challenging, and based on clinical characteristics, there are four types of histoplasmosis infections, namely the asymptomatic (95%), acute pulmonary, chronic pulmonary, and finally, the disseminated variant, which is a rare form [10]. Disseminated histoplasmosis affects the reticuloendothelial system by invading several organs, such as the liver, spleen, pancreas, or intestines. Generally presenting symptoms tend to be nonspecific and diverse including fever, weight loss, anorexia, malaise, cough, abdominal pain, and diarrhea. The most common symptoms of disseminated histoplasmosis are fever (89.1%), respiratory symptoms (38.1%), and weight loss (37.4%) and the most common physical findings include splenomegaly, hepatomegaly, lymphadenopathy, and our patient presented with fever, nonproductive cough, generalized malaise, lymphadenopathy on examination, and hepato-splenomegaly on imaging [11].

Histoplasmosis diagnosis is a combination of clinical presentation and laboratory diagnosis made by utilizing either fungal culture, fungal stains, serologic tests for antibodies, and urine antigen detection. Fungal culture appears to be the gold standard for diagnosis; however, its long turnover of two to four weeks and low sensitivity are limitations. Serologic testing detects H. capsulatum antibodies rapidly but is also associated with false positive results, especially in immunocompromised patients. However, the Histoplasma urine antigen rapidly diagnoses histoplasmosis, as shown in our patient, who had a positive urine antigen despite negative serologic testing [12].

When a patient is diagnosed with disseminated histoplasmosis, amphotericin B is considered the treatment of choice, and a decrease in fever is usually noticed within the first week of commencement of therapy. Treatment can subsequently be changed to itraconazole within three to 14 days because of the systemic toxicities associated with extended amphotericin B use. In our case, our patient completed 10 days of amphotericin B therapy before switching to Itraconazole. For patients with immunosuppression due to HIV, it is essential to commence antiretroviral therapy in addition to histoplasmosis management [13].

## Conclusions

Histoplasmosis suspicion can be challenging as other diseases, such as TB and sarcoidosis, and endemic systemic mycosis, such as coccidioidomycosis, can mimic it. The lack of adequate testing modalities in endemic areas and the limitations of currently available testing methodologies also constitute a barrier to the timely diagnosis and management of suspected histoplasmosis cases. There is a need for further research on testing limitations in histoplasmosis and the institution of a global surveillance and treatment program, especially among immunocompromised and at-risk persons from endemic countries.

Due to the high global burden of disease and high mortality rate among individuals with disseminated histoplasmosis, clinicians should exhibit a high suspicion index among immunosuppressed patients with HIV along with fever of unknown etiology and among migrants from endemic areas who present with non-nonspecific symptoms to ensure timely diagnosis and management.
